# The impact of remote work on worker’s health

**DOI:** 10.47626/1679-4435-2022-828

**Published:** 2023-02-13

**Authors:** Alexandra Lima Roque, Silvia Maria Pimenta, Rita Ribeiro, Ana Branquinho, Teresa Martinho Valente, Elvira Rodriguez Perea, Juan Carlos Fonnegra

**Affiliations:** 1 Serviço de Saúde Ocupacional, Centro Hospitalar Lisboa Ocidental, Lisboa, Lisboa, Portugal

**Keywords:** pandemics, lifestyle, venous thrombosis, coronavirus infections, pandemias, estilo de vida, trombose venosa, infecções por coronavírus

## Abstract

Remote work was brought to the forefront with the arrival of the COVID-19 public health emergency. Although there is no evidence of a direct cause-and-effect relationship between venous disease and work, the current medical consensus is that work can severely intensify its progression. Here, we report the case of a worker at a financial institution, who had been working remotely for around one year and had stopped exercising regularly for the same period. In January 2021, he presented intense pain and marked edema in the soleus area of the right lower limb, which prompted a visit to the emergency department. Laboratory analyses showed slight increases in d-dimer (720 ng/mL) and C-reactive protein (5 mg/dL) levels. A venous Doppler ultrasound of the lower limbs revealed the presence of an occlusive thrombus in the right soleus veins, reaching the right popliteal vein with associated venous dilation. The diagnosis of right, popliteal-distal acute deep vein thrombosis was thus reached. It is clearly impossible to change some of the risk factors of chronic venous insufficiency; however, other aspects such as obesity and working conditions can be the object of preventive actions that generate changes. We thus highlight the importance and possibility of a multidisciplinary approach to this theme, which could evolve into the establishment of a protocol for the prevention and treatment of venous diseases according to each job position.

## Abstract (pt)

O *home office* ganhou um papel de destaque com a chegada da emergência de saúde pública relativa à COVID-19. Embora não haja evidência da relação direta de causa e efeito de doença venosa com o trabalho, existe consenso atual, na opinião médica, de que o trabalho pode agravar seriamente o seu desenvolvimento. É relatado o caso de um profissional de instituição bancária, em teletrabalho há cerca de 1 ano, momento em que suspendeu também a prática de exercício físico. Em janeiro de 2021, iniciou quadro de dor intensa e edema acentuado na região solear do membro inferior direito, motivo pelo qual recorreu ao Serviço de Urgência. No estudo analítico, foram destacadas ligeiras elevações dos D-dímeros (720 ng/mL) e proteína C-reativa (5 mg/dL). A ecografia com estudo de Doppler venoso dos membros inferiores revelou presença de trombo oclusivo nas veias soleares direitas com extensão à veia poplítea direita com dilatação venosa associada. Admitiu-se, então, o diagnóstico de trombose venosa profunda poplíteo-distal direita de características agudas. É evidente que é impossível modificar alguns dos fatores de risco para a insuficiência venosa crônica; porém, outros fatores como obesidade e condições de trabalho podem ser alvo de ações preventivas que gerem modificações. Assim, ressaltam-se a importância e a possibilidade da abordagem multidisciplinar ao tema, o que poderia evoluir para o estabelecimento de um protocolo de prevenção e tratamento de doenças venosas em função do posto de trabalho executado.

## INTRODUCTION

COVID-19 is a multisystem infection caused by SARS-CoV-2; it is potentially severe, has a high transmissibility, and is globally distributed. Ever since the identification of the first cases, the virus continued to disseminate, reaching a dimension (in case numbers and geographic distribution) that determined its classification as a pandemic by the World Health Organization (WHO) in March 11, 2020. According to the WHO, this pandemic has recently infected more than 20 million people in more than 200 countries worldwide and resulted in more than 730,000 deaths.^1^

The COVID-19 pandemic influenced and changed many aspects of our daily lives, such as eating and physical exercise habits. We had a higher tendency of consuming foods that were not as healthy, as the availability of fresh foods and vegetables was frequently limited. Moreover, regarding the constant lockdown and quarantine measures imposed by the countries’ policies, the global population found itself obligated to stay at home for long periods, which led to an exponential increase in physical inactivity. Although these measures are highly recommendable to mitigate dissemination of the disease, they may induce unhealthy behaviors such as a sedentary lifestyle, with most individuals adhering to social distancing measures by working or studying remotely or, in other cases, self-isolating under mandatory quarantines.^2^

This was the moment when remote work reached a leading role. The COVID-19 public health emergency had workers working from their homes for the first time and even more systematically in the near future. Remote work, also known as telework, quickly became the new reality, as requires in the pandemic context in which we lived. Remote work can be defined as the use of information and communication technologies (ICTs)-smartphones and tablet, laptop, and desktop computers-with the aim of working outside of the employer’s premises.^3^ A growing trend of this work modality has been seen in recent years, possibly owing to the advances of ICTs and the advantages associated with remote work.

Although there is no evidence of a direct cause-and-effect relationship between venous disease and work, the current medical consensus is that work can severely intensify its progression. It is known that immobility, namely working in a sitting position, has been considered a risk factor for a first event of venous thrombosis. The disease results from the formation of blood clots in the veins and, more rarely, in the arteries of the lower limbs due to difficulties in blood circulation. It was formerly known as “traveler’s thrombosis,” because it happened mainly in those who had to travel long distances. Today, is has been demonstrated to occur during day-to-day activities such as working.^4^

The following clinical case report describes the history of a worker at a financial institution who had been working remotely for around one year when the initial clinical picture of deep vein thrombosis (DVT) occurred. Considering that the real impact of this pandemic on the physical and psychological health of the global population is still unknown, this study stands as a warning for possible diseases of increasing importance in a very near future.

## CASE REPORT

This case concerns a worker at a financial institution, aged 62 years, male, with a history of arterial hypertension, dyslipidemia, benign prostate hyperplasia, and lumbar and thoracic pathology, with unknown smoking habits. His usual medication regimen was 50 mg atenolol once a day, 10 mg rosuvastatin once a day, and 0.4 mg tamsulosin once a day. At the moment of the report, he was performing corporate client management tasks at a financial institution and was working remotely in the pandemic context since March 2020; he had also stopped exercising regularly for the same period.

In January 2021, he began presenting intense pain and marked edema in the soleus area of the right lower limb, which prompted a visit to the emergency department (ED). On physical examination at the ED, he had an arterial pressure of 140/90 mmHg, a heart rate of 80 bpm, had no fever, and was eupneic. Cardiac and pulmonary auscultations did not reveal alterations. On inspection of the lower limbs, the right calf presented inflammatory signs with generalized non-pitting edema and important functional impairment. An initial laboratory investigation did not reveal alterations in the total blood count, prothrombin time (PT), or activated partial thromboplastin time (aPTT), which were within normal levels. However, d-dimer values were slightly increased (720 ng/mL), as well as C-reactive protein levels (5 mg/dL). A venous Doppler ultrasound of the lower limbs revealed the presence of an occlusive thrombus in the right soleus veins, reaching the right popliteal vein with associated venous dilation. The permeability of the other popliteal and femoral axes did not present signs of acute or chronic obstructive or reflux lesions. The diagnosis of right, popliteal-distal acute DVT was thus reached.

Considering the DVT diagnosis, therapy with low molecular weight heparin (LMWH) was started. On the 2nd day, oral anticoagulant (OA) therapy was started and LMWH was interrupted on the 7th day after reaching the International Normalized Ratio (INR) value.^2,3^ The assessment after 3 months of treatment revealed only a slight edema of the affected limb, and the control Doppler ultrasound revealed almost complete recanalization of the thrombus.

The patient had a short-term partial disability certificate for around 1 month and a half and had a pre-return-to-work medical consultation and examination at the occupational health department. After being examined by an occupational doctor, he was considered conditionally fit for work, with the following recommendations: having 30-minute pauses during the morning and afternoon periods for doing short walks; wearing elastic compression stockings; and resuming regular physical activity as soon as possible. He should be re-evaluated in up to 6 months.

## DISCUSSION

Remote work is not a new work modality; nevertheless, it has become broadly employed with the mobility restrictions caused by the COVID-19 pandemic. Even though companies have returned to normality, many of them will maintain this work modality for longer or even indefinitly.^5^

The loss of a routine, the promotion of remote work regimens, and remaining at home and abandoning physical activity habits (consequently increasing sedentary lifestyles) are some of the factors involved in changes to day-to-day lives that can trigger the appearance or worsening of venous disease complaints, such as tingling, pain, aches, cramps, and legs that feel heavy, swollen, and/or tired. Therefore, the higher occurrence of much longer sitting periods contributes to increased risks of circulatory problems, mainly DVT.^6^

DVT is one of the most prevalent medical conditions, with a current estimated global incidence of 5 cases every 10,000 people. This condition, along with pulmonary thromboembolism (PTE), its complication, is one of the main causes of death among hospitalized patients in the United States, where around 300,000 patients are estimated to die annually due to these pathologies.^7^

DVT results from the occlusion, by a blood clot, of venous blood flow in the deep circulation of the limbs. In most cases, it happens in the lower limbs, more frequently in the deep veins located in the ankle and at venous valves (where higher venous stasis happens due to anatomical reasons) or vessel convergence areas. Prolonged immobilization during work induces compression of the popliteal vein, leading to reduced blood flow in the lower leg and a higher tendency of clot formation. Typical symptoms include leg pain and edema, redness, increased temperature, and in some cases, complications such as PTE. This complication occurs when a blood clot fragment is released in the bloodstream and blocks a blood vessel in the lungs.^7,8^

It is clearly impossible to change some of the risk factors of chronic venous insufficiency, such as age or family history; however, other factors such as obesity and working conditions can be the object of preventive actions that lead to changes. In this context, occupational health professionals have a fundamental role. It would be advisable to implement prophylactic measures, systematically identifying risk factors and elaborating feasible suggestions to improve working conditions. Moreover, it is important to highlight that these professionals are responsible for designing strategies for the prevention and treatment of this disease, even though it is not currently considered an occupational disease.^4^

However, when specifically considering the remote work modality, the perspective of occupational health remains limited by the lack of knowledge on actual working conditions, generating an “imaginative” approach of the work demands at each worker’s home, which are certainly very diverse. It is even harder to approach other health risks for these workers considering the psychosocial risk factors that are not remotely limited to cognitive and emotional constraints and mental burden, but also comprehend decisive organizational aspects such as the number of hours worked or working hours and lack of control over breaks (if they exist).^3^ Nevertheless, occupational doctors as well as general and family doctors should remind their patients of how important it is to be active at home and to maintain crucial measures in the prevention and control of chronic venous disease, considering the context of each person.

We highlight the general measures that are common to most remote work professions: (1) avoid standing or sitting for long periods (particularly with crossed legs), since the blood weight and lack of exercise favor blood stagnation in the veins. Strategies that can be adopted include standing up or walking around the house while at the phone or during commercial or meeting breaks, and walking around the house as much as possible when performing household chores; (2) have a regular walking routine, which is the most beneficial activity for venous circulation; (3) have a complete, varied, and balanced diet, in order to prevent obstipation and overweight, which are responsible for increased venous blood pressure; (4) do “cycling” movements before sleep and lift the feet 10 to 15 centimeters from the bed, which increases blood circulation during sleep; and (5) wear elastic compression stockings of adequate degrees of compression according to the severity of the disease, especially for those who sit or stand up for long periods.^6,7^

This clinical case report is a good example of the changes observed in the workplace, both in work modalities and the resulting pathologies. While working from home used to be a remote and secondary hypothesis for generating income, today it represents an important impact on the finance and daily lives of most of the global population. These measures have thus become even more essential and necessary and should assume the form of medical prescriptions, since despite all therapy advances and improvements, prevention is still the best therapeutic strategy.^2,6,8^

We thus highlight the importance and possibility of a multidisciplinary approach to this theme, which could evolve into the establishment of a protocol for the prevention and treatment of venous diseases according to the job position held by an employee. This would be the beginning of a process of identifying this disorder as an occupational disease, which would consequently contribute to a conceptual reformulation of occupational benefits.^9,10^
